# The membrane-cytoskeletal protein 4.1N is involved in the process of cell adhesion, migration and invasion of breast cancer cells

**DOI:** 10.3892/etm.2012.653

**Published:** 2012-08-03

**Authors:** ZHENYU JI, XIAOFANG SHI, XIN LIU, YU SHI, QINGQING ZHOU, XILONG LIU, LI LI, XIANG JI, YANFENG GAO, YUANMING QI, QIAOZHEN KANG

**Affiliations:** 1 Department of Bioengineering, Zhengzhou University, Zhengzhou 450001;; 2 Henan Academy of Medical and Pharmaceutical Sciences, Zhengzhou University, Zhengzhou 450052;; 3 Basic Medical College of Zhengzhou University, Zhengzhou 450001, P.R. China

**Keywords:** breast cancer, protein 4.1 superfamily, protein 4.1N, metastasis

## Abstract

Protein 4.1N belongs to the protein 4.1 superfamily that links transmembrane proteins to the actin cytoskeleton. Recent evidence has shown that protein 4.1 is important in tumor suppression. However, the functions of 4.1N in the metastasis of breast cancer are largely unknown. In the present study, MCF-7, T-47D and MDA-MB-231 breast cancer cell lines with various metastatic abilities were employed. Protein 4.1N was found to be expressed in poorly metastatic MCF-7 and middle metastatic T-47D cell lines, and was predominantly associated with cell-cell junctions. However, no 4.1N expression was detected in the highly metastatic MDA-MB-231 cells. Moreover, re-expression of 4.1N in MDA-MB-231 cells inhibited cell adhesion, migration and invasion. The results suggest that protein 4.1N is a negative regulator of cell metastasis in breast cancer.

## Introduction

Breast cancer is considered to be one of the most commonly diagnosed cancers in women and metastasis remains the major cause of cancer-related mortality (1,2). Metastasis is a multi-step process that includes the detachment of cancer cells from the primary site, migration and invasion of tumor cells into the blood or lymphatic vessels, as well as motility and invasion into the new target tissue (3). Cytoskeletal reorganization and cell movement underlie all the metastatic events, as well as the disruption of adhesiveness.

Protein 4.1 is a cytoskeletal protein most extensively studied in red blood cells (4.1R) where it stabilizes the spectrin-actin network and anchors it to the plasma membrane. 4.1N was originally designated as a neuronal homologue of the erythrocyte 4.1. The cytoskeletal protein 4.1 family comprising 4.1R (4), 4.1B (5), 4.1G (6) and 4.1N (7) was detected in various cell types and tissues. However, the functions of protein 4.1 in non-erythroid cells are not as clear as the functions of 4.1R in the mature red blood cells. The protein 4.1 family is characterized by the presence of an N-terminal membrane binding domain (MBD). The MBD of the 4.1 protein is closely associated, in sequence and in structure, to the N-terminal domains of ezrin, radixin and moesin (the ERM proteins), and is, therefore, commonly referred to as the FERM domain (8–10). A molecule with a FERM domain at the N-terminus belongs to the protein 4.1 superfamily. Over 40 members have been identified in this superfamily (11).

Although the membrane-binding domains of the proteins of the ERM and 4.1 families share a high degree of sequence homology, and a marked difference in their functions has been detected. For example, it has been observed that ezrin promotes cell growth and may be key in tumor metastasis (12,13), while increasing evidence suggests that members of the 4.1 protein family act as tumor suppressors (14–17). Loss of 4.1B was observed in a variety of human tumors, including meningiomas, non-small cell lung cancers and breast carcinomas (18–22). Protein 4.1R is also involved in brain tumors (16). In a screen for genes involved in breast cancer metastasis, 4.1N expression was found to be absent in highly metastatic breast cancer MDA-MB-231 cells, whereas poorly metastatic cells, 4.1N was expressed and predominantly associated with cell-cell junctions. Thus, we re-introduced protein 4.1N in highly metastatic breast cancer MDA-MB-231 cells to validate its potential tumor metastatic suppressive function.

## Materials and methods

### Cell lines and culture conditions

The human breast cancer cell lines, MCF-7, T-47D and MDA-MB-231 (all provided by the American Type Culture Collection, ATCC, Manassas, VA, USA), were used in the present study. MCF-7 is a low metastatic breast cancer cell line, while T-47D is a middle and MDA-MB-231 a highly metastatic breast cancer cell line. The three cell lines were grown in DMEM medium (Gibco, Invitrogen, Carlsbad, CA, USA) supplemented with 10% fetal bovine serum (FBS) at 37°C in a humidified atmosphere containing 5% CO_2_. The cells were subcultured every 2–3 days to maintain exponential growth.

### Western blot analysis

Western blot analysis was performed for the cell lines to confirm the presence or absence of the protein 4.1N. Cell lysates were prepared in RIPA buffer (50 mM Tris, 150 mM NaCl, 0.1% SDS, 0.5% sodium deoxycholate, 1% NP40) with protease inhibitors. Total proteins were quantified by the Bradford method using the BCA protein assay kit according to the manufacturer’s instructions. Samples were boiled at 100°C in loading buffer for 10 min and 30 μg protein from each sample were electrophoresed on a 10% SDS-polyacrylamide gel (SDS-PAGE) and then transferred to PVDF membranes by semi-dry transfer apparatus (Bio-Rad, Hercules, CA, USA). The membranes were blocked in TBST (25 mM Tris-HCl, pH 7.5, 137 mM NaCl, 2.7 mM KCl and 0.05% Tween-20) with 5% fat-free milk for 1 h at 37°C, and then incubated with the primary antibodies (rabbit anti-4.1N and rabbit anti-GFP, provided by Dr Xiuli An from the New York Blood Center, Melville, NY, USA) in blocking buffer overnight at 4°C. After washing three times with TBST, the membranes were incubated with secondary antibody (goat anti-rabbit conjugated with HRP; Jackson ImmunoResearch Laboratories, Inc., West Grove, PA, USA) for 1 h at room temperature and finally exposed to Kodak BioMax Film using Super ECL Detection Reagent. The total protein loading on gel was reconfirmed by blotting with an antibody against GAPDH (Abcam, Cambridge, MA, USA). Protein quantification was performed by ImageJ software.

### Immunocytochemistry

Immunocytochemistry was used to detect the location of protein 4.1N. Cells were grown on 12-mm chamber slides for 48 h and fixed immediately in 1% polyoxymethylene for 15 min, then permeabilized with 0.1% Triton X-100 in 0.25% PFA/PBS for another 15 min. Cells were blocked using 0.1% Triton X-100 in 0.25% PFA/PBS with 10% horse serum for 30 min, then incubated with primary antibody (anti-4.1N) diluted in antibody buffer for 60 min at room temperature. Goat anti-rabbit IgG secondary fluorescein Alexa Fluor™ 488-labeled antibody was added and incubated at room temperature for 40 min. Stained cells were visualized using appropriate filters on a Nikon Eclipse E800M microscope and imaged using a Sony Cats Eye Digital Photo Camera and Imaging System.

### Cell transfection and screening

MDA-MB-231 cells were seeded into 6-well plates at a density of 5×10^5^ cells/well and maintained in a 37°C incubator to obtain 80–90% confluence. Cell transfection was performed using Lipofectamine^®^ 2000 transfection reagent according to the manufacturer’s instructions. Briefly, 4 μg of plasmids pEGFP-4.1N (provided by Dr Xiuli An with sequencing identification being performed in our laboratory) or pEGFP-3C and 6 μl of Lipofectamine^®^ 2000 (Invitrogen) were gently mixed with 250 μl of serum-free DMEM without antibiotics for a 10-min incubation at room temperature. The two mixtures were combined and kept at room temperature for another 20 min. The complex was then added to the cells. After incubation at 37°C for 24 h, G418 (800 μg/ml; Invitrogen) was applied to stably screen and isolate the resistant colonies. MDA-MB-231 cells transfected with pEGFP-4.1N and pEGFP-C3 were designated as EGFP-4.1N/MDA-MB-231 and EGFP/MDA-MB-231. Stable transfectant clones with high protein 4.1N expression were identified by western blot analysis and observed under fluoro-scope microscopy.

### Cell adhesion assay

Cell adhesion was evaluated according to the modified methods described by Charboneau *et al* (22). Briefly, the 96-well tissue culture plates were coated with 5 μg fibronectin (Fn) for each well and incubated at 4°C overnight. Cells (1×10^4^) suspended in DMEM containing 0.1% BSA were dispensed into each well of the 96-well plates and incubated with 5% CO_2_ at 37°C for 60 min, then gently washed twice with PBS to remove the unattached cells. After fixing with 3.8% PFA for 15 min, the cells were incubated with 0.2% crystal violet solution to stain the cells for 1 h at room temperature and then washed twice with distilled water. Dye extraction was performed by adding 100 μl of 10% acetic acid solution and agitating for 10 min. The absorbance was measured at 570 nm. Each assay was performed in triplicate and repeated at least twice in independent experiments.

### Wound-healing assay

Cells were grown to confluence on culture plates and a wound was made in the monolayer with a sterile P200 pipette tip (∼0.5 mm in width). The recovery of these monolayer cells was dependent on cell proliferation and migration during wound-healing. After wounding, the medium and debris were removed by washing three times with PBS, and fresh medium was added to the wells. Images of the wound were captured at 0 and 20 h after wounding to observe the changes in migration. A mean wound area was determined using ImageJ software and the average area of wound closure was calculated.

### Cell migration and invasion assay

Cell migration assays were performed using a Transwell chamber (8.0-μm pore size PET inserts; Becton Dickinson, Franklin Lakes, NJ, USA). The bottom chamber was coated with 10 μg/ml Fn diluted in PBS and incubated at 37°C overnight. Cells (5x10^4^/well) suspended in 200 μl of DMEM with 0.1% bovine serum albumin were seeded into the upper chamber either uncoated (for migration assay) or coated (for invasion assay) with Matrigel. Cells were allowed to migrate over 16 h and the cells in the bottom chamber were fixed with 3.8% PFA, followed by staining with 500 μl of 0.2% crystal violet solution for 2 h at room temperature. The migrated cells were counted under bright field microscopy and photographed. The experiment was performed twice with each sample in triplicate and cell counting was performed in five randomly selected fields.

### Statistical analysis

Data were analyzed with the software package SPSS 12.0 (SPSS, Chicago, IL, USA). P<0.05 was considered to indicate a statistically significant result.

## Results

### Expression and the cellular location of the protein 4.1N in breast cancer cells

**Figure 1:**
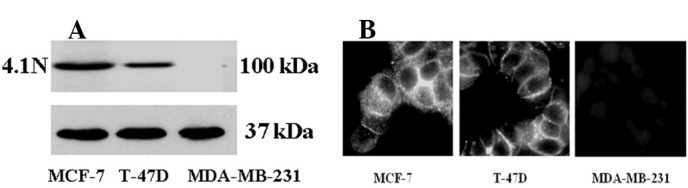
Expression and subcellular localization of the protein 4.1N in breast cancer cells. (A) Western blot analysis shows the difference at the protein expression level in three different metastatic cell lines. GAPDH was used as a loading control. (B) Immunocytochemical staining of breast cancer cell lines. Cells were stained with the 4.1N antibody and visualized with the Alexa 488 conjugated anti-rabbit secondary antibody. Protein 4.1N is shown to concentrate at regions of cell-cell contact.

Immunoblotting analysis was first performed to examine the expression level of protein 4.1N in three human breast cancer cell lines with various meta-static abilities. The results demonstrated that protein 4.1N is expressed in the low and middle metastatic MCF-7 and T-47D cell lines, respectively, whereas the protein was not expressed in the highly metastatic MDA-MB-231 cells (Fig. 1A). Immunocytochemistry was used to detect the cellular location of protein 4.1N in breast cancer cell lines with 4.1N antibody. The results showed that 4.1N was mainly expressed in the cell-cell junctions in the low and middle metastatic cells. However, 4.1N was not expressed in MDA-MB-231 cells (Fig. 1B). To investigate the roles of 4.1N in the metastasis of human breast cancer cells, the MDA-MB-231 cell line was selected for transfection and additional study.

### Stable transfection of 4.1N

**Figure 2:**
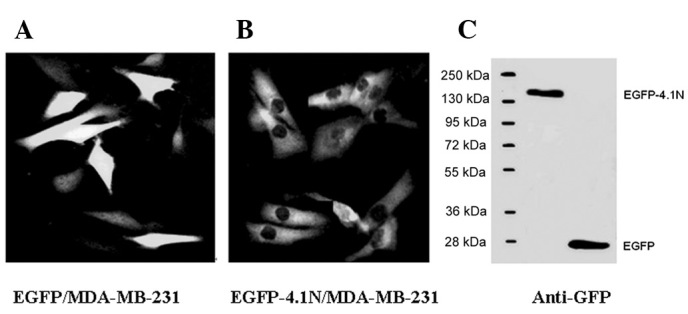
Expression of protein 4.1N in MDA-MB-231 cells after transfection. (A) Fluorescence imaging of EGFP in MDA-MB-231 cells transfected with pEGFP-C3 (magnification, x200). (B) Fluorescence imaging of MDA-MB-231 cells transfected with pEGFP-4.1N. Fusion protein in transfected cells was mainly located in the cytoplasm. (C) The expression of EGFP-4.1N or EGFP was confirmed by western blot analysis using anti-EGFP antibody. Whole cell lysates were separated by 10% SDS-PAGE, followed by immuno-blotting with anti-EGFP antibody (dilution, 1:5,000).

Based on screening by western blot analysis, transfection was conducted using MDA-MB-231 cells with pEGFP-4.1N and an empty pEGFP-C3 vector plasmid was selected for mock transfectant. After the 14-day selection using 800 μg/ml of G418, stable cell clones were obtained and pooled populations of clones were selected to avoid clone variation. Stable transfectant clones with a high protein 4.1N expression were identified by western blot analysis and observed under a fluoroscope microscope. Fluorescence microscopy demonstrated that EGFP is located throughout the cell in diffuse green fluorescence imaging (Fig. 2A), and the ectogenic EGFP-4.1N protein was mainly localized in the cytoplasm (Fig. 2B). Western blot analysis confirmed that EGFP-4.1N fusion protein was highly expressed in MDA-MB-231 cells (Fig. 2C).

### Effect of protein 4.1N on cell adhesion

**Figure 3:**
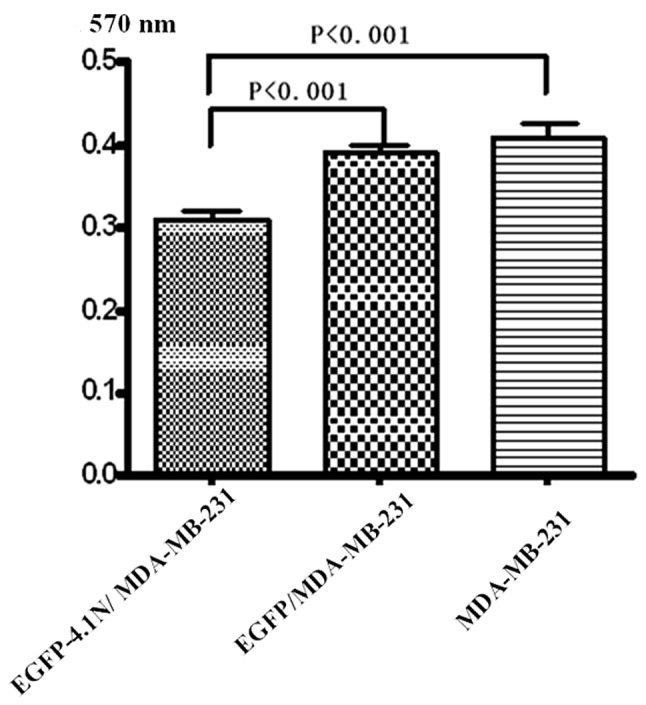
Effect of protein 4.1N transfection on cell adhesion of MDA-MB-231 cells. The number of cells seeded in fibronectin-coated wells was determined by MTT assay. Bars, SDs.

Protein 4.1N localizes to the sub-plasma membrane and, similar to its family members, acts as a linker protein between the cytoskeleton and the plasma membrane. Thus, this protein is capable of modulating tumor cell capacity to adhere to various extracellular matrices, potentially through interaction with transmembrane proteins and organization of the underlying cytoskeleton. The effects of the protein 4.1N on cell adhesion were examined. Results showed that the inhibition of EGFP-4.1N/MDA-MB-231 cell adhesion ability after incubating cells for 60 min in 96-well plates coated with Fn (Fig. 3). A significant difference of cell growth was observed in 4.1N-transfected cells (P<0.001), while no difference was found between EGFP/MDA-MB-231 and MDA-MB-231 cells, suggesting that protein 4.1N modulated MDA-MB-231 cell capacity to adhere to Fn.

### Effect of protein 4.1N on cell migration

**Figure 4:**
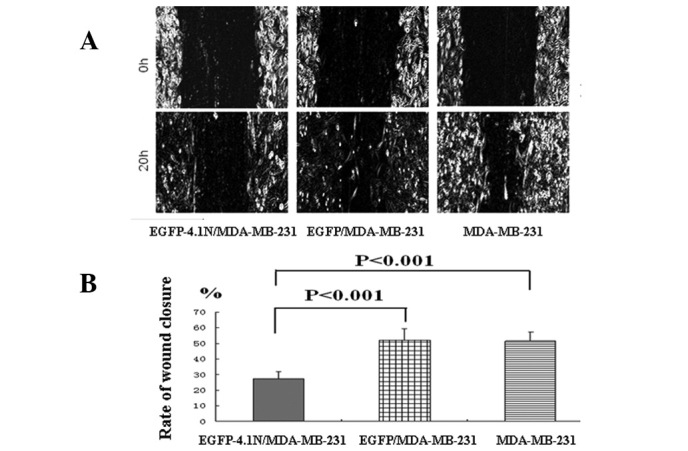
Expression of protein 4.1N attenuates cell migration in the Transwell assay. (A) Cell migration through membrane inserts was analyzed after a 16-h incubation using a modified Transwell assay. Cells that penetrated to the lower surface of the membrane were fixed and stained (magnification, x200). (B) Mean cell counts from at least 10 fields and from three separate experiments are shown. 4.1N transfectants had a significantly reduced migration ability compared to empty vector transfectants and control cells (P<0.001). Bars, SDs.

Wound-healing and Transwell assays were used to investigate whether 4.1N is important in the regulation of cell migration in MDA-MB-231 cells. In the wound-healing assay, images were captured at 0 and 20 h to observe the changes in migration (Fig. 4A). The percentage of wound area closure at 20 h of wound-healing was then calculated (Fig. 4B). EGFP-4.1N/MDA-MB-231 cells had a low wound-healing rate when compared to those of EGFP/MDA-MB-231 and MDA-MB-231 cells (P<0.001).

### Effect of protein 4.1N on cell invasion

**Figure 5:**
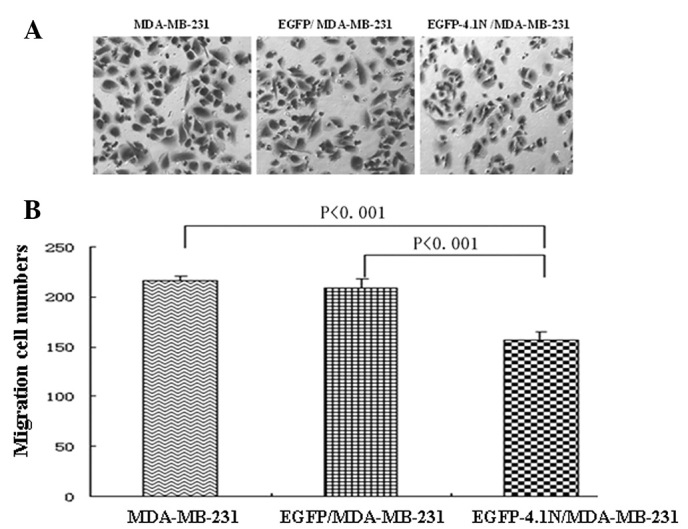
Expression of protein 4.1N affects cell invasion. (A) Cell invasion through Matrigel basement membrane extract was analyzed using a modified Transwell assay. Cells that invaded the lower surface of the membrane were fixed and stained (magnification, x200). (B) Mean cell counts from at least 10 fields and three separate experiments are shown. The number of invaded cells of EGFP-4.1N/MDA-MB-231 (89.3±6.0) was markedly fewer than those of EGFP/MDA-MB-231 and MDA-MB-231 cells (163.3±9.1 and 166.8±6.9; P<0.001, respectively).

To evaluate the role of protein 4.1N in MDA-MB-231 cell invasion, the ability of cells to permeate through a reconstituted basement membrane barrier (Matrigel) was tested using the Transwell assay. EGFP-4.1N/MDA-MB-231 cells (89.3±6.0) showed significantly reduced invasiveness as compared with EGFP/MDA-MB-231 (163.3±9.1) and MDA-MB-231 cells (166.8±6.0; P<0.001; Fig. 5), indicating that the expression of 4.1N in MDA-MB-231 cells is associated with a reduced invasive ability.

## Discussion

Protein 4.1N was first identified in mouse embryonic neurons at the earliest stage of differentiation (7). Both 4.1R and 4.1N are known to have various splice isoforms. The mouse 4.1N predominant isoform in brain is 135 kDa and a smaller 100-kDa isoform was identified in peripheral tissues. By analogy with the roles of 4.1R in red blood cells, 4.1N was considered to confer stability and plasticity to the neuronal membrane via interactions with multiple binding partners, including integral membrane receptors and membrane-associated guanylate kinases. Previously, investigators identified several proteins that interact with 4.1N which possess essential roles in cell proliferation, adhesion and signaling transduction. Typical examples of such proteins include NuMA, PIKE, NECL1 and AMPA receptor subunit GluR1 (23–27). Members of the protein 4.1 family that link transmembrane proteins to the actin cytoskeleton have been demonstrated as tumor suppressors. 4.1B/DAL-1 was originally identified as a protein whose expression was reduced in human non-small cell carcinomas (14). Subsequent studies have shown that the downregulation of 4.1B/DAL-1 occurs across many different tumor cell types including brain, breast, prostate, kidney and sarcoma (28,29). Our previous studies have demonstrated the complete loss of 4.1N expression in 30% of colon cancer samples (28/94), particularly in the poorly differentiated cancers (unpublished data). In the present study, an anti-4.1N-specific antibody was used to detect 4.1N expression and subcellular localization in breast cancer cell lines with various metastatic abilities. Western blot analysis and immunofluorescent results revealed that the 100-kDa protein 4.1N was expressed and mainly located at the cell-cell junctions in the poorly metastatic cell line MCF-7 and middle metastatic cell line T-47D, whereas no protein 4.1N expression was found in highly metastatic MDA-MB-231 cells. The reintroduction of protein 4.1N by transfection with the pEGFP-4.1N plasmid attenuated MDA-MB-231 cell adhesion, migration and invasion, suggesting 4.1N involvement during tumor progression.

Metastasis is a multi-step process including the detachment of cancer cells from the primary site, migration and invasion of tumor cells into the blood or lymphatic vessels, motility and invasion into the new target tissue. Each step creates one or more physiological barriers to the spread of malignant cells. Tumor cells usually have to overcome all of the barriers including altered adhesiveness, increased motility and invasive capacity to successfully proceed to metastasis. Since the adhesion of tumor cells is considered as a key step in the invasive processes of metastatic tumor cells, the effects of 4.1N on cell adhesion were examined. One of the observations was that the reintroduction of 4.1N into MDA-MB-231 cells inhibited cell adhesion, an observation consistent with the function of NF2, a member of the protein 4.1 superfamily. In rat schwannoma cells, NF2 expression transiently reduces cell attachment to Fn, with levels returning to normal after 3 h (30). However, reintroduction of DAL-1 (active segment of 4.1B) into MCF-7 cells increased cell attachment on all extracellular matrix proteins as measured at 1 h using similar short-term adhesion assays (22). NF2 and DAL-1 differentially affect cell adhesion and this is consistent with reports that DAL-1 does not bind to actin (29). Although the details of protein 4.1N cell adhesion inhibition require additional investigation, the data indicate that cells may have an increased transfection ability in the absence of 4.1N.

Tumor cell migration and invasion through the basement membranes are key steps in the multi-stage process that leads to metastatic formation. In the present study, EGFP-4.1N/MDA-MB-231 cells showed significantly decreased cell migration and invasion compared to the mock cells. This is the first evidence suggesting that 4.1N is involved in breast cancer metastasis. 4.1B reportedly acts as a metastasis suppressor since its loss supports a reorganization of the F-actin cytoskeleton and concomitant enhanced cell motility, both of which are likely to be important in metastasis (17). 4.1N potentially serves as an inhibitor of migration and invasion by restoring the membrane cytoskeleton.

In conclusion, this is the first study where the expression of the membrane-cytoskeletal protein 4.1N is associated with breast cancer metastasis. This study verifies that a 100-kDa 4.1N is crucial in cell adhesion, migration and invasion in breast cancer cells.

## Abbreviations:

MBD: membrane binding domain
ERM: ezrin, radixin and moesin
FERM: 4.1 protein-ezrin-radixin-moesin homology
